# Routine laboratory testing in hemodialysis: how frequently is it needed?

**DOI:** 10.1186/s12882-022-02971-9

**Published:** 2022-10-27

**Authors:** Claudia Chidiac, Dania Chelala, Dany Nassar, Chadia Beaini, Hiba Azar, Serge Finianos, Celine Boueri, Jenny Hawi, Ibrahim Abdo, Mabel Aoun

**Affiliations:** 1 Department of Internal Medicine, Faculty of Medicine, Saint-Joseph University, Beirut, Lebanon; 2 Nephrology Department, Faculty of Medicine, Saint-Joseph University, Beirut, Lebanon; 3 Nephrology Department, Bellevue Medical Center, Mansourieh, Lebanon; 4 Nephrology Department, Saint-George Hospital, Ajaltoun, Lebanon; 5 Nephrology Department, Hotel Dieu de France Hospital, Beirut, Lebanon

**Keywords:** Laboratory test, Hemodialysis, Test frequency, Treatment, Anemia, CKD-MBD

## Abstract

**Background:**

Hemodialysis patients are followed by routine laboratory testing. There is uncertainty whether these tests always lead to a change in decision-making. This study aims to discover the number of yearly interventions/changes in prescription based on these tests and depict the group of patients who would benefit from reduced or increased laboratory blood tests.

**Methods:**

This is a multi-center retrospective study that included patients on hemodialysis for more than one year. Laboratory data collected included yearly average of hemoglobin, urea reduction ratio (URR), serum phosphate, calcium, potassium, parathormone (PTH), ferritin and transferrin saturation (TSAT); changes in prescription of erythropoietin-stimulating agents (ESAs), intravenous (IV) iron, alfacalcidol, phosphate binders and dialysis parameters were retrieved from medical records. A multivariate regression analysis assessed factors associated with high number of interventions.

**Results:**

A total of 210 hemodialysis patients were included: 62.4% males, 47.1% diabetics. Their median age was 72 (62,78.5) years. Their laboratory parameters were within KDIGO targets. The median number of yearly interventions was 5 (3,7) for ESAs, 4 (2,6) for IV iron, 1 (0,2.25) for phosphate binders, 0 (0,1) for alfacalcidol. Based on the multivariate analysis, patients with higher ferritin, frequent changes in ESA, more changes in alfacalcidol and higher PTH had higher number of prescription’s changes in ESA, IV iron, phosphate binders and alfacalcidol respectively.

**Conclusion:**

While maintaining KDIGO targets, therapeutic interventions following routine laboratory testing did not exceed six times yearly for all parameters. This suggests that a reduced testing frequency in hemodialysis patients is possible without any impact on quality of care. A personalized approach remains safe for hemodialysis patients while reducing the cost. This is very relevant in low-resource settings and during economic crises and needs to be evaluated in prospective studies.

## Background

Hemodialysis is the most common form of renal replacement therapy worldwide. Hemodialysis regimens can differ among patients but chronic hemodialysis patients have usually three sessions per week with each dialysis session lasting three to five hours [1]. They are usually monitored by routine laboratory testing to ensure dialysis adequacy and detect complications associated with chronic kidney disease stage 5 on dialysis (CKD stage 5D) such as anemia, mineral bone disorders and electrolytes’ disturbances. The best practice worldwide suggests to draw a monthly blood test for these patients. Nevertheless, this routine is not based on solid evidence [2].

The 2017 Kidney Disease: Improving Global Outcomes (KDIGO) guideline on the management of chronic kidney disease-mineral bone disorder (CKD-MBD) suggests measuring serum calcium and serum phosphate every one to three months and serum parathyroid hormone (PTH) every three to six months. In CKD stage 5D patients, laboratory parameters should fall within CKD-MBD KDIGO targets: maintaining serum PTH two to nine times the top margin of normal, avoiding hypercalcemia and lowering serum phosphate toward the normal range [3]. In the absence of robust scientific evidence, KDIGO also recommends monitoring hemoglobin (Hb) every month and both ferritin and transferrin saturation (TSAT) every three months [4]. KDOQI anemia guidelines recommend in patients on chronic hemodialysis a Hb of 11-12 g/dL (not to exceed 13 g/dL) and administration of intravenous iron (IV iron) as long as TSAT < 20% and ferritin < 200 ng/ml [1]. The KDIGO anemia guidelines are also aligned with KDOQI and suggest that Hb does not exceed 11.5 g/dL but they recommend intravenous iron (IV iron) be given as far as ferritin < 500 ng/mL and TSAT < 30% [4].

Several studies tried to determine the best frequency of laboratory sampling in hemodialysis patients in order to maintain patients’ parameters within guidelines’ targets. Gaweda et al. measured Hb weekly for a better anemia management [5]. On the other hand, Greenberg et al. showed that patients monitored monthly instead of quarterly for PTH reached better KDOQI target PTH values [6]. However, Yokoyama et al. found no benefit in a more frequent measurement when serum calcium, phosphate and PTH were in their target values [7]. Most recently, a Canadian study by Silver et al. revealed that blood testing every six weeks instead of every four weeks was sufficient and was not associated with a change in anemia and mineral bone disease targets [8].

There is uncertainty whether the costly regular tests often lead to a change in the decision-making of nephrologists. Our study aims to find out the yearly number of interventions/changes in prescription based on these tests and to depict the specific group of patients who would benefit from reduced testing.

## Materials and methods

### Study design and participants

This a multi-center retrospective study that included patients on hemodialysis for more than twelve consecutive months between February 2012 and February 2021. The study was conducted in three Lebanese dialysis units: Hôtel-Dieu de France (HDF), Saint-Georges Ajaltoun Hospital (HSGA) and Bellevue Medical Center (BMC). The eight nephrologists working in these units managed patients according to the KDIGO guidelines. We excluded from the study patients on dialysis for less than one year, those aged less than 18 years old and any patient who was not tested regularly as per the national guidelines of the country. The Lebanese national guidelines for hemodialysis facilities require monthly measurement of Hb, electrolytes, urea reduction ratio (URR), serum phosphate, serum calcium and every four months PTH, ferritin and TSAT [9].

### Data collection

The data collection was based on the medical records present in each dialysis unit. It included age, gender, cause of ESKD, dialysis vintage (in months), inhibitors of renin angiotensin aldosterone system (RAASi). Comorbidities included diabetes, hypertension, cancer, coronary artery disease (CAD), hepatitis B or C. The laboratory parameters Hb, URR, PTH, TSAT, ferritin, serum potassium, calcium and phosphate were collected from the last year of follow-up. For each patient, twelve values of Hb, URR, serum potassium, phosphate and calcium were summed and divided by twelve; three values of PTH, TSAT and ferritin were summed and divided by three. Over the same year, changes in prescription of erythropoietin stimulating agents (ESAs), IV iron, alfacalcidol, cinacalcet, kayexalate, phosphate binders (PB), and of dialysis parameters were collected.

### Measurements

The laboratory parameters including Hb, platelets, urea, potassium, ferritin, TSAT (iron/TIBC), calcium, phosphate, PTH and serum albumin were measured using standard techniques in the three hospitals. PTH levels of the HDF hospital were multiplied by two because they were measured using the third-generation technique. Blood samples were taken immediately before starting dialysis during the mid-week session.

### Statistical analysis

Statistical analyses were performed using the Statistical Package for the Social Sciences, Version 24.0 (SPSS Inc.-IBM corp., Armonk, NY, USA). Continuous data with normal distribution were reported as mean and standard deviation (SD) and as median and interquartile (IQR) if non-normally distributed. Continuous variables such as number of interventions per year were reported as both means and medians if normally distributed because the median was used to divide the sample into two groups for the logistic regression. Categorical data were reported as numbers and percentages. Mann Whitney test, independent t-test, Chi Square test and Fischer’s Exact test were used to compare two groups of interventions. In order to analyze the factors associated with high number of interventions per year, we divided the sample into two groups based on the median. A multivariate logistic regression analysis was performed to assess factors associated with high frequency of interventions; the model included all significant values from the bivariate analysis. *P-*value < 0.05 was considered as statistically significant.

## Results

### Patient characteristics

A total of 210 hemodialysis patients were included: 88 patients from HDF, 84 from HSGA and 38 from BMC (Table 1). The median age of patients at the time of the study was 72 years old (62,78.5). The median age of patients at dialysis initiation was 68 (59, 75). The median dialysis vintage was 34 months (20.75, 66), 62.4% of patients were males and 47.1% had diabetes. Their laboratory parameters were within KDIGO targets (highlighted in Table 1). The median number of interventions per year (changes of prescription) was 5 (3,7) for ESAs, 4 (2,6) for IV iron, 1 (0,2.25) for any phosphate binder and 0 (0,1) for alfacalcidol.

**Table 1:** General characteristics of all patients

	Total N = 210
**Age (years), median (IQR)**	72 (62, 78.25)
**Sex, M/F (%)**	131/79 (62.4/37.6)
**Dialysis vintage (months), median (IQR)**	34 (20.75, 66)
**Nephropathy** -Diabetic -Nephrosclerosis -Glomerulonephritis -Autosomal dominant polycystic kidney disease (ADPKD) -Unknown etiology -Chronic tubulointerstitial nephritis	89 (42.4) 35 (16.7) 26 (12.4) 15 (7.1) 32 (15.2) 13 (6.2)
**Diabetes, n(%)**	99 (47.1)
**Hypertension, n(%)**	201 (95.7)
**Smoking status, n(%)**	80 (38.1)
**CAD, n(%)**	82 (39)
**Cancer, n(%)**	28 (13.3)
**Hepatitis B, n(%)**	0 (0)
**Hepatitis C, n(%)**	0 (0)
**Hemoglobin over one year, median (IQR)** **Hemoglobin ≥ 11 g/dL, n(%)** **Hemoglobin ≥ 10.5 g/dL, n(%)**	11.27 (10.78, 11.78) 132 (62.9) 176 (83.8)
**Ferritin over one year, ng/mL, mean ± SD**	533.89 ± 258.26
**TSAT over one year, median (IQR)** **TSAT ≥ 20%, n(%)**	0.26 (0.21, 0.31) 172 (81.9)
**Serum calcium over one year, mg/dL, mean ± SD**	9.01 ± 0.48
**Serum phosphate over one year, mg/dL, mean ± SD** **Serum phosphate < 5 mg/dL, n(%)**	4.4 ± 1.1 175 (83.3)
**PTH, over one year, pg/mL, median (IQR)**	232.4 (129.7, 413.2)
**URR over one year, mean ± SD**	0.75 ± 0.05
**Serum potassium over one year, median (IQR)** **Serum potassium > 6meq/L, n(%)**	5.11 (4.73, 5.45) 13 (6.2)
**RAAS inhibitors intake, n(%)**	23 (11)
**Monthly dose of ESA over one year, UI, median (IQR)**	21833.5 (14958.25, 40833.0)
**Monthly dose of IV Iron ampoules (100 mg) over one year, mean ± SD**	1.15 ± 1.07
**Weekly dose of alfacalcidol, µg, median (IQR)**	1 (0, 2)
**Calcium-based phosphate binder (CBB), n(%)**	172 (81.9)
**Non-calcium-based phosphate binder (NCBB), n(%)**	94 (44.8)
**Yearly number of interventions in ESA prescription, mean ± SD** **Median (IQR)**	4.63 ± 2.62 5 (3, 7)
**Yearly number of interventions in IV iron prescription, mean ± SD** **Median (IQR)**	4.25 ± 0.1 4 (2, 6)
**Yearly number of interventions in alfacalcidol prescription, median (IQR)**	0 (0, 1)
**Yearly number of interventions in CBB prescription, median (IQR)**	0 (0, 2)
**Yearly number of interventions in NCBB prescription, median (IQR)**	0 (0, 1)
**Yearly number of interventions in any phosphate binder prescription, median (IQR)**	1 (0, 2.25)
**Yearly number of interventions in cinacalcet prescription, median (IQR)**	0 (0, 0)
**Yearly number of interventions in kayexalate prescription, median (IQR)**	0 (0, 0)
**Yearly number of interventions in potassium dialysate prescription, median (IQR)**	0 (0, 1)
**Yearly number of interventions in session duration prescription, median (IQR)**	0 (0, 0)
**Yearly number of interventions in filter surface prescription, median (IQR)**	0 (0, 0)

### Clinical outcome 1: number of ESA interventions per year

Two groups of patients were compared based on the median of ESA interventions per year, those who had a change of prescription 5 times a year or less and those who had > 5 interventions per year (Table 2). Those who had lower hemoglobin levels (median 11 g/dL), higher ferritin levels (mean 633.8 ng/mL), higher TSAT levels (median 28%) and who were in the first three years of dialysis (median 31 months) needed changes of ESA prescription in ESA above 5 times a year.

Based on the multivariate logistic regression analysis, ferritin, iron dose and number of interventions for iron per year were associated with a higher frequency of ESA interventions per year (Table 3).

When comparing patients with autosomal dominant polycystic kidney disease (ADPKD) to the rest of patients, they had significantly less interventions for ESA (p = 0.024) with significant lower doses of ESA monthly (p = 0.009) and higher levels of hemoglobin (p = 0.004).

**Table 2:** Comparison of two groups of ESA interventions

	With ≤ 5 interventions per year for ESA n = 120	With > 5 interventions per year for ESA n = 90	*P*-value
**Age, median (IQR)**	72 (61, 78)	72 (63, 79.3)	0.422*
**Sex, M/F (%)**	77/44 (64.2/35.8)	54/36 (60/40)	0.537**
**Dialysis vintage (months), Median (IQR)**	40 (25, 72)	31 (18, 49.3)	0.011*
**Diabetes, n(%)**	54 (45)	45 (50)	0.473**
**Hypertension, n(%)**	116 (96.7)	85 (94.4)	0.502***
**Smoking status, n(%)**	50 (41.7)	30 (33.3)	0.218**
**CAD, n(%)**	53 (44.2)	29 (32.6)	0.09**
**Cancer, n(%)**	14 (11.7)	14 (15.6)	0.412**
**RAAS inhibitors intake, n(%)**	12 (10)	11 (12.2)	0.61**
**Monthly dose of ESA, Median (IQR)**	22333.5 (10,250, 45624.8)	21,333 (17249.8, 37,500)	0.853*
**Monthly dose of IV iron, Mean ± SD**	1.04 ± 1.02	1.29 ± 1.13	0.005****
**Yearly number of interventions in ESA prescription** **Mean ± SD**	2.8 ± 1.7	7.1 ± 1.1	< 0.001****
**Yearly number of interventions in IV iron prescription** **Mean ± SD**	3.7 ± 2.7	5.0 ± 2.1	< 0.001****
**Hemoglobin over one year, Median (IQR)**	11.5 (10.9, 11.9)	11.0 (10.6, 11.4)	< 0.001*
**Ferritin over one year, Mean ± SD**	459.8 ± 260.9	633.8 ± 219.2	< 0.001****
**TSAT over one year, Median (IQR)**	0.25 (0.20, 0.29)	0.28 (0.22, 0.35)	0.002*
**Serum calcium over one year, Mean ± SD**	9.0 ± 0.5	9.0 ± 0.4	0.770****
**Serum phosphate over one year, Mean ± SD**	4.5 ± 1.04	4.3 ± 1.1	0.217****
**PTH, over one year, Median (IQR)**	270.0 (166.8, 509.0)	175.4 (101.4, 312.7)	0.001*
**URR over one year**, **Mean ± SD**	0.74 ± 0.05	0.77 ± 0.05	0.002****
**Serum potassium over one year, Median (IQR)**	5.1 (4.8, 5.6)	5.1 (4.7, 5.4)	0.215*

*Mann Whitney test and **Chi Square test ***Fischer’s Exact test ****Independent t-test; P-value < 0.05 is statistically significant

**Table 3:** Multivariate logistic regression analysis of factors associated with a change in ESA prescription more than 5 times per year

	OR	95% CI	*P*-value
**Dialysis vintage**	0.99	0.99; 1.01	0.544
**ESA dose**	1.00	1.00; 1.00	0.430
**Iron dose**	1.59	1.12; 2.25	0.010
**Number of changes in iron prescription per year**	1.23	1.08; 1.41	0.003
**Hemoglobin level**	0.91	0.62; 1.32	0.608
**Ferritin level**	1.004	1.002;1.005	< 0.001
**TSAT percentage**	0.98	0.75; 1.26	0.855
**PTH level**	0.99	0.99; 1.00	0.048
**URR percentage**	1.06	0.99; 1.14	0.113

*Note. OR, odds ratio; 95%CI, 95% Confidence Interval; TSAT, transferrin saturation; iron dose in ampoules of 100 mg; dialysis vintage in months; p-value < 0.05 is statistically significant. We included in this model all variables with P-value < 0.05 in the bivariate analysis*

### Clinical outcome 2: number of IV iron interventions per year

For IV iron, we compared patients who had ≤ 4 interventions per year to patients with > 4 interventions per year (Table 4). Those who had lower hemoglobin levels (median 11.1 g/dL), higher mean yearly modifications of ESA (5.5 ± 2.3) and who were older (median 74 years old) needed changes of prescription in IV iron above 4 times a year.

Based on the multivariate logistic regression analysis, older age and higher number of changes in ESA prescriptions were associated with a higher frequency of IV iron prescriptions’ modifications per year (Table 5).

**Table 4:** Comparison of two groups of IV iron interventions

	With ≤ 4 interventions per year and less for Iron n = 110	With > 4 interventions per year for Iron n = 100	*p*-value
**Age, median (IQR)**	68 (61, 78)	74 (65, 79.8)	0.021*
**Sex, M/F (%)**	72/38 (65.5/34.5)	59/41 (59/41)	0.335**
**Dialysis vintage (months), median (IQR)**	36 (25, 67)	32.5 (19, 64.3)	0.234*
**Diabetes, n(%)**	53 (48.2)	46 (46)	0.752**
**Hypertension, n(%)**	106 (96.4)	95 (95)	0.739****
**Smoking status, n(%)**	37 (33.6)	43 (43)	0.163**
**CAD, n(%)**	50 (45.5)	32 (32)	0.052**
**Cancer, n(%)**	18 (16.4)	10 (10)	0.175**
**Monthly dose of ESA, median (IQR)**	21,500 (14958.3, 44750.3)	22,000 (14658.8, 37,875)	0.612*
**Monthly dose of IV iron, mean ± SD**	1.1 ± 1.04	1.2 ± 1.1	0.874***
**Yearly number of interventions in ESA prescription** **Mean ± SD**	3.8 ± 2.6	5.5 ± 2.3	< 0.001***
**Yearly number of interventions in IV iron prescription** **Mean ± SD**	2.2 ± 1.3	6.5 ± 1.4	< 0.001***
**Hemoglobin over one year, median (IQR)**	11.3 (10.8, 11.9)	11.1 (10.8, 11.6)	0.039*
**Ferritin over one year, Mean ± SD**	508.2 ± 287.5	561.9 ± 220.1	0.134***
**TSAT over one year, median (IQR)**	0.25 (0.21, 0.30)	0.26 (0.21, 0.32)	0.423*
**URR over one year, mean ± SD**	0.75 ± 0.05	0.76 ± 0.05	0.316***
**Serum potassium over one year, median (IQR)**	5.2 (4.8, 5.6)	5.1 (4.6, 5.4)	0.120*

*Mann Whitney test, **Chi Square test, ***Independent t-test ****Fischer’s Exact test; P-value < 0.05 is statistically significant

**Table 5:** Multivariate logistic regression analysis of factors associated with a change in IV iron prescription more than four times per year

	OR	95% CI	*P*-value
**Age**	1.03	1.003; 1.05	0.027
**Hemoglobin level**	0.93	0.69; 1.24	0.632
**Number of changes in ESA prescription per year**	1.31	1.15; 1.49	< 0.001

*Note. OR, odds ratio; 95%CI, 95% Confidence Interval; P-value < 0.05 is statistically significant. We included in this model all variables with P-value < 0.05 in the bivariate analysis*

### Clinical outcome 3: number of phosphate binders’ interventions per year

Patients on calcium-based phosphate binder (CBB) or non-calcium-based phosphate binder (NCBB) were divided into 2 groups: those with ≤ 1 interventions per year against those with > 1 intervention per year (Table 6). Those who had lower dialysis vintage (median 31 months), who were smokers and who had higher mean yearly modifications of alfacalcidol, higher hemoglobin, PTH and phosphate needed changes of prescription in phosphate binders more than once per year.

Based on the multivariate logistic regression analysis, smokers and higher number of changes in alfacalcidol prescriptions were associated with a higher frequency of phosphate binder prescriptions’ modifications per year (Table 7).

**Table 6:** Comparison of two groups of phosphate binders’ interventions

	With ≤ 1 interventions per year for phosphate binders n = 133	With > 1 interventions per year for phosphate binders n = 77	*P*-value
**Age, median (IQR)**	72 (62, 80)	70 (62, 77)	0.284*
**Sex, M/F (%)**	86/47 (64.7/35.3)	45/32 (58.4/41.6)	0.370**
**Dialysis vintage (months), median (IQR)**	38 (25, 67)	31 (17.5, 53.5)	0.031*
**Diabetes, n(%)**	59 (44.4)	40 (51.9)	0.289**
**Hypertension, n(%)**	125 (94.9)	76 (98.7)	0.159***
**Smoking status, n(%)**	43 (32.3)	37 (48.1)	0.024**
**CAD, n(%)**	57 (42.9)	25 (32.9)	0.156**
**Cancer, n(%)**	17 (12.8)	11 (14.3)	0.757**
**Yearly number of interventions in alfacalcidol prescription**, **median (IQR)**	0 (0, 1)	0 (0, 2)	0.021*
**Hemoglobin over one year, median (IQR)**	11.1 (10.6, 11.7)	11.4 (10.9, 11.8)	0.021*
**PTH over one year, median (IQR)**	211.6 (97.5, 374.9)	278.0 (174.5, 463.2)	0.007*
**Serum calcium over one year, Mean ± SD**	9.02 ± 0.43	8.99 ± 0.57	0.811****
**Serum phosphate over one year, Mean ± SD**	4.29 ± 1.13	4.59 ± 0.95	0.045****
**URR over one year, Mean ± SD**	0.76 ± 0.05	0.75 ± 0.05	0.099****

*Mann Whitney test and **Chi Square test ***Fischer’s Exact test ****Independent t-test; P-value < 0.05 is statistically significant

**Table 7:** Multivariate logistic regression analysis of factors associated with a change in phosphate binders more than once per year

	OR	95% CI	*P*-value
**Dialysis vintage**	0.99	0.99; 1.01	0.426
**Smoking status**	2.10	1.15; 3.86	0.017
**Yearly number of interventions in alfacalcidol prescription**	1.49	1.08; 2.06	0.015
**Hemoglobin level**	1.29	0.99; 1.69	0.057
**PTH level**	1.001	1.000; 1.002	0.199
**Serum phosphate**	1.18	0.88; 1.59	0.273

*Note. OR, odds ratio; 95%CI, 95% Confidence Interval; P-value < 0.05 is statistically significant. We included in this model all variables with p value < 0.05 in the bivariate analysis*

### Clinical outcome 4: number of alfacalcidol interventions per year

A comparison was done for changes in prescription of alfacalcidol by dividing patients into two groups: with no interventions versus ≥ 1 intervention per year (Table 8). Those who were younger (median age 67 years) and those who had higher PTH levels needed at least one yearly modification in phosphate binders’ prescription.

**Table 8:** Comparison of two groups of alfacalcidol interventions

	With no interventions per year for Alfacalcidol n = 121	With ≥ 1 interventions per year for Alfacalcidol n = 89	*P*-value
**Age, median (IQR)**	74 (64.5, 81)	67 (60.5, 75)	< 0.001*
**Sex, M/F (%)**	80/41 (66.1/33.9)	51/38 (57.3/42.7)	0.193**
**Dialysis vintage, median (IQR)**	34 (20, 66)	35 (21, 62)	0.880*
**Diabetes, n(%)**	61 (50.4)	38 (42.7)	0.268**
**Hypertension, n(%)**	116 (95.9)	85 (95.5)	0.999***
**Smoking status, n(%)**	47 (38.8)	33 (37.1)	0.795**
**CAD, n(%)**	46 (38)	36 (40.9)	0.672**
**Cancer, n(%)**	15 (12.4)	13 (14.6)	0.642**
**Yearly number of interventions in phosphate binders’ prescription**, **median (IQR)**	1 (0, 2)	1 (0, 3)	0.199*
**Hb over one year, median (IQR)**	11.3 (10.7, 11.8)	11.3 (10.8, 11.6)	0.881*
**PTH over one year, median (IQR)**	207.6 (99.8, 299.2)	338.5 (171.0, 555.7)	< 0.001*
**Serum calcium over one year, Mean ± SD**	9.02 ± 0.49	8.99 ± 0.48	0.678****
**Serum phosphate over one year, Mean ± SD**	4.35 ± 1.08	4.48 ± 1.08	0.362****
**URR over one year, Mean ± SD**	0.76 ± 0.05	0.75 ± 0.05	0.070****

*Mann Whitney test and **Chi Square test ***Fischer’s Exact test ****Independent t-test; P-value < 0.05 is statistically significant

## Discussion

Our study showed that routine monthly blood tests in hemodialysis patients were not always needed to modify prescriptions of medications or dialysis parameters. In the majority of our patients, the anemia and CKD-MBD blood parameters were maintained within the KDIGO targets despite the lack of a regular or monthly change in prescriptions. The median number of interventions per year targeting anemia were 5 and 4 for ESA and IV iron respectively. These changes in prescription occurred less frequently compared to the routine of monthly hemoglobin testing and triannual iron parameters’ testing. This suggests that reducing hemoglobin monitoring to every two months can be feasible in the majority of patients. Furthermore, the need for more frequent interventions in those with a lower hemoglobin level at a median of 11 g/d, higher ferritin levels at a median of 634 ng/mL and higher TSAT at a median of 28% suggests a personalized approach in this category of patients. Our study did not include C-Reactive Protein measurement to assess infection or inflammation in these patients with low hemoglobin and high ferritin; however, the high median TSAT level is not in favor of functional iron deficiency and makes inflammation less plausible. Another factor that was found associated with higher interventions for ESA in our study is dialysis vintage. This may result from the lower response to ESA when patients have been on chronic dialysis for longer period of time. This has been reported by Gaweda et al. who described an increase in ESA dose prescription with the increase of dialysis vintage from 4.6 to 7.3 years [10]. Regarding iron prescription, our regression analyses’ results showed that more interventions on IV iron were significantly associated with older age and high number of ESA changes but not associated with ferritin or TSAT levels. The fact that IV iron prescription change exceeded the number of ferritin dosage per year in our study shows that IV iron prescription was driven as well by hemoglobin levels. It is uncertain whether the iron parameters are needed three times per year or less. To the best of our knowledge, this has not been extensively studied. Very few studies tackled the number of blood tests needed per year to adapt hemodialysis patients’ prescriptions. In 2018 and 2019, two Canadian studies compared routine blood sampling performed every 6 weeks instead of every 4 weeks. They found that hemoglobin and CKD-MBD targets were reached as recommended, and the 4-week testing was not associated with lower risk of death neither cardiovascular events [2, 8]. In a small sample of 49 hemodialysis patients from the United States, Gaweda et al. found that measuring hemoglobin weekly compared to monthly reduces the error of variability and may lead to a better management of anemia [5]. However, they did not compare more prolonged intervals of sampling.

ADPKD is the most common genetic cause of ESKD, with 12.5 million cases worldwide [11, 12]. Usually, ESKD patients suffer from anemia as a consequence of decreased production of erythropoietin (EPO) by damaged kidneys. However, this does not apply to ADPKD [12]. Several studies showed a serum EPO level in ADPKD twofold higher than in ESKD of other causes along with higher hemoglobin and hematocrit values. These findings were attributable to the production of EPO by renal cysts [11, 13, 14]. Our results concur with all these studies showing higher hemoglobin values in ADPKD patients, less interventions and lower doses of ESA. Shah et al. demonstrated that higher hemoglobin reached in PKD patients was associated with a better survival with infrequent ESA administration in contrast to a higher mortality with frequent ESA administration [11]. Hence, ADPKD patients can be less frequently tested for hemoglobin than with patients with ESKD of other causes.

Interestingly, our study has shown a reduced need for measurement of PTH, serum calcium and serum phosphate. The median number of interventions ranged between 0 and 1 per year. Serum calcium in particular has not shown to affect any of the changes in prescription of alfacalcidol or phosphate binders in our sample of chronic hemodialysis patients. Curiously, smokers in our study needed more interventions with phosphate binders; this finding is aligned with Santos et al. who demonstrated higher phosphate levels in smokers [15]. In our study, higher levels of serum phosphate and PTH were significantly associated with more frequent changes in phosphate binders’ prescription but not exceeding six times per year. We also found higher PTH levels in the group of younger patients, which concurs well with the study of Yu et al. [16]. Our findings suggest that reducing calcium, phosphate monitoring to every two or three months and PTH to twice per year would be sufficient to support the physician’s decision-making regarding CKD-MBD targets in hemodialysis patients. It is noteworthy that reaching a more stable PTH level within KDOQI targets would need more frequent monthly measurements as it was shown by Greenberg et al. [6]. However, Yokohama et al. demonstrated that there is only a benefit from frequent monitoring when serum calcium, phosphate and PTH exceed their targeted values [7]. It is also important to mention that phosphate and PTH were measured in the morning in approximately half of our patients and in the afternoon in the other half. Several studies have reported a circadian variation of these two parameters [17, 18] but we did not compare the morning and afternoon groups.

In chronic hemodialysis patients, dialysis adequacy is usually assessed either by the URR or the Kt/V with a target of at least 65% and 1.2 respectively [19, 20]. A higher dose of HD is an important indicator of clinical performance and can lead to better survival as shown by Held et al. [21]. In our study, the median of URR over one year was within target, equal to 0.75 (0.72, 0.79) and no significant yearly change was noted for the session duration or the filter surface. According to the European Best Practice Guidelines (EBPG) as well as the National Kidney Foundation/Kidney Disease Outcomes Quality Initiative (NFK/DOQI), the dose of hemodialysis should be measured at least monthly [22, 23]. Nonetheless, Couchoud et al. found that 40% of dialysis units were checking urea removal less frequently than once per month [20]. We suggest to reduce the measurement of URR to 3 or 4 times per year with a personalized approach to critical patients.

Our study showed very few changes in chronic prescription of potassium dialysate or kayexalate. We found that 6% of our sample had an average serum potassium over one year above 6 meq/L. This is very close to the literature where hyperkalemia was estimated at 10% in hemodialysis patients [24]. Potassium control in CKD patients is essential since hyperkalemia is associated with the risk of cardiac arrhythmias and sudden cardiac death [25]. It is well-known as well that hyperkalemia in hemodialysis mainly results from high potassium diet [26]. Therefore, the most common intervention following hyperkalemia is promoting awareness about the patient’s potassium intake. This kind of intervention was not included in our data collection. Therefore, we suggest monitoring serum potassium every two months in hemodialysis patients except in cases of frequent hyperkalemia where monthly monitoring is preferred.

This study has several strengths. To the best of our knowledge, it is the first study in Lebanon and one of the rare studies worldwide evaluating the frequency of laboratory testing in patients on chronic hemodialysis. In addition, patients with different characteristics were evaluated. A prolonged interval of laboratory monitoring could decrease healthcare costs especially in low-income countries. The major limitation of our study is the retrospective design that confines conclusions to data collected. Data on dietary counseling would have been important when assessing interventions towards phosphate and potassium levels and this needs to be addressed in future prospective studies. The lack of data on acute supplemental sessions, transfusions and acute blood tests drawn on top of routine laboratory tests prevents us from drawing conclusions about the total number of tests per year; however, the aim of our study was to evaluate the frequency of chronic routine testing and not the tests taken in acute settings.

## Conclusion

In conclusion, the median of interventions per year based on routine laboratory testing did not exceed 6 times for all parameters in this study. This suggests that the routine testing in CKD stage 5D patients can be reduced without any impact on the quality of care. Based on these findings, we suggest a testing for hemoglobin, serum calcium and phosphate every two months and for URR and PTH twice a year. A more specific patient-related approach remains safe and could minimize laboratory costs especially in low-resource settings. Prospective and larger studies are required to determine the best interval of testing in chronic hemodialysis patients on a global scale.

## Data Availability

The datasets used and/or analysed during the current study are available from the corresponding author on reasonable request.

## List of abbreviations

**CKD**: chronic kidney disease
**CKD-MBD**: chronic kidney disease-mineral bone disorder.
**KDIGO**: Kidney Disease:Improving Global Outcomes.
**TSAT**: transferrin saturation.
**PTH**: parathyroid hormone.
**ESA**: erythropoietin stimulating agent.
**IV iron**: intravenous iron.

## References

[1] National Kidney Foundation. KDOQI Clinical Practice Guideline for Hemodialysis Adequacy: 2015 update. *Am J Kidney Dis*. 2015 Nov;66(5):884–930. 10.1053/j.ajkd.2015.07.015. Erratum in: *Am J Kidney Dis*. 2016 Mar;67(3):534. PMID: 26498416. 10.1053/j.ajkd.2015.07.015 26498416

[2] Thomas A, Silver SA, Perl J, Freeman M, Slater JJ, Nash DM, Vinegar M, McArthur E, Garg AX, Harel Z, Chanchlani R, Zappitelli M, Iliescu E, Kitchlu A, Blum D, Beaubien-Souligny W, Wald R (2020). The Frequency of Routine Blood Sampling and Patient Outcomes Among Maintenance Hemodialysis Recipients. Am J Kidney Dis.

[3] Kidney Disease: Improving Global Outcomes (KDIGO) CKD-MBD Update Work Group (2017). KDIGO 2017 Clinical Practice Guideline Update for the Diagnosis, Evaluation, Prevention, and Treatment of Chronic Kidney Disease-Mineral and Bone Disorder (CKD-MBD). Kidney Int Suppl.

[4] Kidney Disease (2012). Improving Global Outcomes (KDIGO) Anemia Work Group KDIGO clinical practice guideline for anemia in chronic kidney disease. Kidney Int Suppl.

[5] Gaweda AE, Nathanson BH, Jacobs AA, Aronoff GR, Germain MJ, Brier ME (2010). Determining optimum hemoglobin sampling for anemia management from every-treatment data. Clin J Am Soc Nephrol CJASN.

[6] Greenberg S, Gadde S, Pagala M, Greenberg M, Shneyderman I, Janga K (2011). Optimal frequency of parathyroid hormone monitoring in chronic hemodialysis patients. Clin Nephrol.

[7] Yokoyama K, Kurita N, Fukuma S, Akizawa T, Fukagawa M, Onishi Y, Kurokawa K, Fukuhara S. Frequent monitoring of mineral metabolism in hemodialysis patients with secondary hyperparathyroidism: associations with achievement of treatment goals and with adjustments in therapy. *Nephrol Dial Transplant* 2017 Mar 1;32(3):534–541. 10.1093/ndt/gfw020. PMID: 26945054; PMCID: PMC5837642. 10.1093/ndt/gfw020 PMC5837642 26945054

[8] Silver SA, Alaryni A, Alghamdi A, Digby G, Wald R, Iliescu E (2019). Routine Laboratory Testing Every 4 Versus Every 6 Weeks for Patients on Maintenance Hemodialysis: A Quality Improvement Project. Am J Kidney Dis.

[9] Aoun M, Makkouk J, Ammar W. Ultrapure water in haemodialysis: a step towards better quality in Lebanon. *East Mediterr Health J Rev Sante Mediterr Orient Al-Majallah Al-Sihhiyah Li-Sharq Al-Mutawassit*. 2019;25(2):134–141. 10.26719/emhj.18.032. 10.26719/emhj.18.032 30942478

[10] Gaweda AE, Jacobs AA, Aronoff GR, Brier ME (2018). Individualized anemia management in a dialysis facility - long-term utility as a single-center quality improvement experience. Clin Nephrol.

[11] Shah A, Molnar MZ, Lukowsky LR, Zaritsky JJ, Kovesdy CP, Kalantar-Zadeh K (2012). Hemoglobin level and survival in hemodialysis patients with polycystic kidney disease and the role of administered erythropoietin. Am J Hematol.

[12] Colbert GB, Elrggal ME, Gaur L, Lerma EV (2020). Update and review of adult polycystic kidney disease. Dis–Mon DM.

[13] Eckardt KU, Möllmann M, Neumann R (1989). Erythropoietin in polycystic kidneys. J Clin Invest.

[14] Chandra M, Miller ME, Garcia JF, Mossey RT, McVicar M (1985). Serum immunoreactive erythropoietin levels in patients with polycystic kidney disease as compared with other hemodialysis patients. Nephron.

[15] Santos GDD, Elias RM, Dalboni MA, Silva GVD, Moysés RMA (2019). Chronic kidney disease patients who smoke have higher serum phosphorus. J Bras Nefrol.

[16] Yu Y, Diao Z, Wang Y, Zhou P, Ding R, Liu W. Hemodialysis patients with low serum parathyroid hormone levels have a poorer prognosis than those with secondary hyperparathyroidism. Ther Adv Endocrinol Metab. 2020 Sep 21;11:2042018820958322. 10.1177/2042018820958322. PMID: 33014329; PMCID: PMC7513009. 10.1177/2042018820958322 PMC7513009 33014329

[17] Trivedi H, Szabo A, Zhao S, Cantor T, Raff H (2015). Circadian variation of mineral and bone parameters in end-stage renal disease. J Nephrol.

[18] Dario KA, Dalboni MA, da Silva BC, Steller Wagner Martins C, de Araújo LKRP, Elias RM, Moysés RMA. Predialysis serum phosphate levels according to hemodialysis shift: Circadian rhythm matters. Hemodial Int. 2021 Jan;25(1):134–136. 10.1111/hdi.12882. Epub 2020 Oct 5. PMID: 33015995. 10.1111/hdi.12882 33015995

[19] Eknoyan G, Beck GJ, Cheung AK (2002). Effect of dialysis dose and membrane flux in maintenance hemodialysis. N Engl J Med.

[20] Couchoud C, Jager KJ, Tomson C, Cabanne JF, Collart F, Finne P, de Francisco A, Frimat L, Garneata L, Leivestad T, Lemaitre V, Limido A, Ots M, Resic H, Stojceva-Taneva O, Kooman J, QUEST working group on dialysis adequacy (2009). Assessment of urea removal in haemodialysis and the impact of the European Best Practice Guidelines. Nephrol Dial Transplant.

[21] Held PJ, Port FK, Wolfe RA (1996). The dose of hemodialysis and patient mortality. Kidney Int.

[22] Pyart R, Magadi W, Steenkamp R, Davenport A (2018). Chapter 6 Adequacy of Haemodialysis in UK Adult Patients in 2016: National and Centre-specific Analyses. Nephron.

[23] Hemodialysis Adequacy 2006 Work Group. Clinical practice guidelines for hemodialysis adequacy, update 2006. *Am J Kidney Dis*. 2006 Jul;48 Suppl 1:S2-90. 10.1053/j.ajkd.2006.03.051. PMID: 16813990. 10.1053/j.ajkd.2006.03.051 16813990

[24] Ahmed J, Weisberg LS (2001). Hyperkalemia in dialysis patients. Semin Dial.

[25] Watanabe R. Hyperkalemia in chronic kidney disease. *Rev Assoc Medica Bras 1992*. 2020;66Suppl 1(Suppl 1):s31-s36. 10.1590/1806-9282.66.S1.31. 10.1590/1806-9282.66.S1.31 31939533

[26] Pani A, Floris M, Rosner MH, Ronco C (2014). Hyperkalemia in hemodialysis patients. Semin Dial.

